# Improving the Chemical Stability of Al Alloy through the Densification of the Alumina Layer Assisted by SiF_6_^2−^ Anion Hydrolysis

**DOI:** 10.3390/nano12081354

**Published:** 2022-04-14

**Authors:** Mosab Kaseem, Burak Dikici, Hongfei Liu

**Affiliations:** 1 Department of Nanotechnology and Advanced Materials Engineering, Sejong University, Seoul 05006, Korea; 2 Department of Metallurgical and Materials Engineering, Ataturk University, Erzurum 25240, Turkey; burakdikici@atauni.edu.tr; 3 Institute of Materials Research and Engineering (IMRE), A*STAR (Agency for Science, Technology and Research), 2 Fusionopolis Way, Singapore 138634, Singapore

**Keywords:** Al alloy, plasma electrolysis, densification, hydrolysis, corrosion, frontier molecular orbital

## Abstract

In this work, a high-density alumina layer with high chemical stability was successfully developed by controlling the hydrolysis of hexafluorosilicate (SiF_6_^2−^) anions through the addition of various concentrations of sodium citrate (SCi) into the electrolyte of plasma electrolysis (PE). To achieve this aim, the substrate samples were anodized in alkaline aluminate–SiF_6_^2−^-based electrolytes with 0, 5, and 10 g/L of SCi. The presence of SCi anions in the electrolyte led to the formation of a thick adsorbed electrochemical double layer (EDL) on the substrate surface. The EDL not only affected the movement of SiF_6_^2−^ anions towards the anode but also influenced their hydrolysis reaction, which in turn led to a controllable sealing of structural defects with the hydrolysis products, namely SiO_2_ and AlF_3_. Among three different oxide layers, the oxide layer obtained from the electrolyte with 5 g/L SCi showed the highest chemical stability in a corrosive solution, which was linked to the fact that a considerable increase in the compactness of the oxide layers was obtained by the incorporation of SiO_2_ and AlF_3_. The mechanism underlying the effects of SCi on triggering the hydrolysis of SiF_6_^2−^ anions and factors affecting chemical stability are discussed based on the experimental data and computational analysis.

## 1. Introduction

Plasma electrolysis (PE) is an electrochemical surface treatment method utilized to modify metallic surfaces. It has been widely explored in academic research and industry [1]. The PE process often operates at high voltages that cause the dielectric breakdown of the initially formed thin layer accompanied by micro-discharges [1,2,3]. However, the porous morphology of the oxide layer made via PE is a significant factor that demands suitable control to obtain a wider range of applications [1]. The surface properties of the oxide layer are affected by many factors related to the electrolyte composition, coating time, current density, frequency, duty cycle, etc. [1]. Among them, the impact of the electrolyte composition on the surface characteristics of the oxide layers has been reported to be significant [1]. For instance, Arunnellaiappan et al. [4], using impedance tests in a 5 wt.% NaCl solution, found that the corrosion of 7075 Al alloy was greatly inhibited by the inert incorporation of CeO_2_ nanoparticles into the alumina layer obtained via PE. Recently, Hussain et al. [5] stated that the co-existence of sodium oxalate (SOx) and sodium citrate (SCi) in the electrolyte was an effective procedure to develop soft plasma discharges during the PE treatment of Al alloy, and a dense layer with improved corrosion resistance was successfully obtained. 

On the other hand, the inclusion of F^−^ anions into the oxide layers is an effective strategy that provides a considerable increment in the chemical stability of PE coatings [6,7,8,9]. By a careful examination of the published literature on PE coating with a fluoride additive, it can be concluded that F^−^ anions are desirable for enhancing corrosion performance. For instance, in one study, undissolved MgF_2_ was generated on an Mg alloy surface; it prevented the excessive dissolution of the substrate and reduced the tendency of pit formation [8]. According to the results reported by Ryu and Hong [9], the addition of KF to alkaline aluminate electrolytes during PE led to the development of a fluorine-enriched inner layer together with high amounts of MgAl_2_O_4_, leading to the enhancement of the corrosion-protection properties of Mg alloy. However, in terms of Al alloy, the addition of F^−^ anions into the electrolyte can be described by complex behavior during the PE process, as reported by Yerokhin et al. [10]. Wang et al. [11] found that the impact of NaF on the chemical stability of PE-treated Al alloy was significant.

In another study, potassium hexafluorosilicate (K_2_SiF_6_)—as an indirect source of Si and F elements—was hydrolyzed in alkaline solutions, leading to the formation of SiO_2_ and F^−^ anions, which exhibited important practical properties [12]. Thus, the observations made in previous studies raise the question of whether it is possible to use complex salts containing fluorine, such as K_2_SiF_6_, to fabricate defect-free coatings via PE, since the hydrolysis of K_2_SiF_6_ would be influenced by the amounts of SCi existing in the electrolyte during PE. Therefore, this work aimed to fabricate a high-density layer on a 6061 alloy to improve its chemical stability using the PE process and controlling the hydrolysis of SiF_6_^2−^ anions with different concentrations of SCi.

## 2. Materials and Methods

### 2.1. Formation of the Oxide Layers via PE

Commercially 6061 Al alloy sheets (25 mm × 20 mm × 4 mm) were utilized as substrates on which the PE coatings were deposited. Before PE, the substrates were polished with SiC emery papers up to 2400 grit, cleaned with acetone, and dried by air. The experimental parameters of current density, frequency, and coating time were controlled to be 100 mA·cm^−2^, 60 Hz, and 300 s, respectively. To examine the effects of SCi concentrations on the hydrolysis of SiF_6_^2−^ anions, several concentrations of SCi (0, 5, and 10 g/L) were included in the electrolyte that contained 1 g/L KOH, 3 g/L NaAlO_2_, and 3 g/L K_2_SiF_6_. Here, S0, S5, and S10 refer to the oxide layers obtained from electrolytes with 0, 5, and 10 g/L SCi, respectively. All reagents were purchased from Sigma-Aldrich (St. Louis, MO, USA) and used as received.

### 2.2. Microstructural and Compositional Analysis

The morphologies and compositions of the samples were characterized by field-emission scanning electron microscopy (SEM-HITACHI, PT-S2800, Hitachi, Japan) linked with energy-dispersive X-ray spectroscopy (EDS). The porosity level in the oxide layers was determined using SEM images taken from at least ten different areas for each condition with the help of the Image J analyzer software. The direct 2D calculation using SEM images was preferred over plausible 3D methods that have been reported previously [2] because of two reasons that could affect the experimental results. First, the application of the techniques mentioned above would likely require the delamination of the coating layer from the substrate, which would significantly damage the inner layer close to the substrate. Second, the porosity results obtained utilizing the 3D techniques might be exaggerated because these methods measure not only the micropores but also the oxide nodules. The phase composition of the coated samples was identified using X-ray diffraction (XRD, X′pert Pro Diffractometer, Philips, The Netherlands) and X-ray photoelectron spectroscopy (XPS, VG Microtech, ESCA 2000, VG Microtech, London, UK).

### 2.3. Electrochemical and Quantum Chemical Computational Analysis

The chemical stability of the samples was evaluated by potentiodynamic polarization (PDP) and electrochemical impedance spectroscopy (EIS). A typical three electrode cell with a Pt plate, Ag/Ag/AgCl, and the tested sample as the counter, reference, and working electrodes, respectively, were used to conduct the electrochemical measurements. PDP tests were conducted from −0.25 V to +0.40 V (vs. open circuit potential (OCP)) at a scan rate of 1 mV/s, while EIS tests were measured from 0.1 Hz to 10^6^ Hz at 10 points/decade using a 10 V RMS AC signal. The samples were left in the corrosive solution (a 3.5 wt.% NaCl) for 6 h to stabilize the OCP and the measurements were conducted at least three times to obtain reliable results. To calculate the quantum chemical parameters of SCi anions, the Chem3D pro 11.0 and Chem3D pro 17.0 software was used [5]. Based on the values of the highest occupied molecular orbital (HOMO) and lowest molecular orbital (LUMO) energies, the electron affinity (*A*), ionization potential (*I*), absolute hardness (*η*), absolute softness (*σ*), Mulliken electronegativity (χ), and the fraction of electron transfer (Δ*N*) were computed.

## 3. Results and Discussion

### 3.1. Morphology and Composition of the Oxide Layers

**Figure 1:**
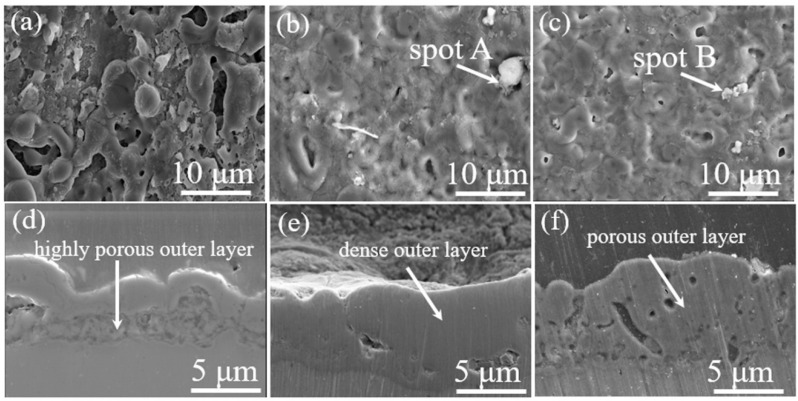
Images of the surface and cross-section for the (**a**,**d**) S0 sample, (**b**,**e**) S5 sample, and (**c**,**f**) S10 sample.

Figure 1a–c presents the changes in the surface morphology of the S0, S5, and S10 samples obtained from electrolytes containing various contents of SCi. As shown in Figure 1a, different levels of micropores and oxide nodules were discovered on the surface of the S0 sample as a result of gas evolution and high energy plasma during discharging activity at the coating/electrolyte interface [1]. The plasma discharges can reach a high temperature of approximately 5800 K, causing the local melting of the adjacent oxide layer. The molten Al will be ejected toward the coating/electrolyte interface to form Al_2_O_3_ via a plasma-assisted chemical reaction [13,14]. However, the sizes of such structural defects were reduced upon the inclusion of SCi in the electrolyte, as shown in Figure 1b,c, suggesting that SCi helped to suppress pore formation due to the evolution of O_2_ gas. Among all samples, the S5 sample was the most compact, as the porosity decreased from 10.41% in S0 to 1.81 and 3.35% with 5 and 10 g/L of SCi in the electrolyte, respectively.

The results of the EDS analysis conducted on the surface of the S0, S5, and S10 samples are tabulated in Table 1. Regardless of the SCi concentrations, the elements found in the oxide layers were Al, O, Si, C, and F. The high temperature that causes the decomposition of SCi during PE is responsible for the appearance of C in the S5 and S10 samples [5]. Interestingly, the amounts of Si and F incorporated into the oxide layer tended to increase with an increase in SCi concentration, even though the three electrolytes had the same concentration of K_2_SiF_6_. This result suggested that SCi promoted the participation of SiF_6_^2−^ anions in the formation of the oxide layers. Moreover, some pores—specifically on the surface of the S5 and S10 samples—were blocked with white particles, as seen in Figure 1b,c. To identify the composition of the white particles, an EDS point analysis was conducted on spot A and spot B in Figure 1b,c. The element compositions in spot A were 14.01% “Si”, and 28.04% “O”, while spot B was composed of 13.56% Si and 26.47% “O”. Thus, the white particles were composed of SiO_2_, as the atomic ratio of Si to O was approximately 0.5.

Figure 1d–f shows the cross-sectional images of the S0, S5, and S10 samples. The thickness of the oxide layers was calculated to be 4.56 ± 1.1, 6.21 ± 0.8, and 7.17 ± 0.7 µm for the S0, S5, and S10 samples, respectively. Thus, the influence of SCi on the thickness of the oxide layer seemed to be significant. Interestingly, the S5 sample was relatively dense compared to the S0 and S10 samples, implying that the presence of 5 g/L of SCi in the electrolyte containing SiF_6_^2−^ ions was beneficial in improving the compactness of not only the inner layer but also the outer porous layer, which would lead to high chemical stability in the corrosive media.

**Figure 2:**
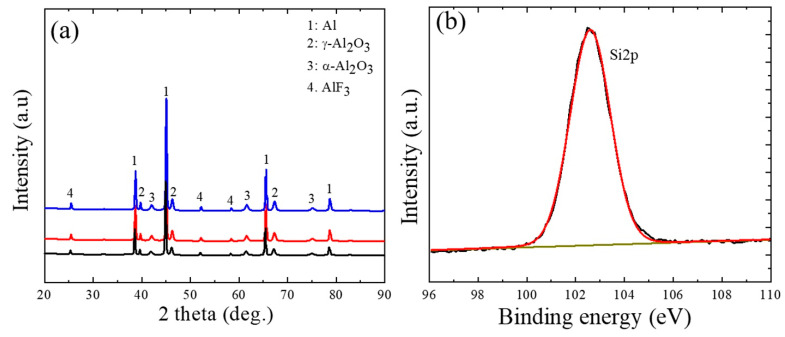
(**a**) XRD patterns for the S0, S5, and S10 samples. (**b**) High-resolution XPS spectrum of Si2p in the S10 sample.

The XRD patterns in Figure 2a showed the presence of γ-Al_2_O_3_, α-Al_2_O_3_, and AlF_3_ in all the samples, regardless of SCi concentration. The formation of γ-Al_2_O_3_ and α-Al_2_O_3_ is related to the reaction between Al^3+^ cations (from the substrate) and O^2−^ anions (from the electrolyte) under fast and slow cooling rates, respectively. On the other hand, the formation of AlF_3_ can be attributed to the hydrolysis of SiF_6_^2−^ anions, as shown in Equation (1) [15].

(1) $$SiF_{6}{}^{2-}+2H_{2}O\to SiO_{2}+4H^{+}+6F^{-}$$

The AlF_3_ is formed by the reaction between the F^−^ anions in Equation (2) and the Al^3+^ cations produced from the substrate (Equation (2)).

(2) $$Al^{3+}+3F^{-}\to AlF_{3}$$

Although the EDS results revealed the presence of Si atoms in the oxide layers, the XRD results did not show any crystal phases of Si, which suggests that the Si-based compounds incorporated into the oxide layers were in the amorphous form. To confirm the incorporation of Si into the oxide layers, the XPS spectrum for Si2p of the S10 sample was analyzed to obtain more information on the composition of the Si compounds in the oxide layer. As shown in Figure 2b, the Si2p spectrum revealed only one peak at approximately 103.2 eV that corresponded to SiO_2_, suggesting that SiO_2_ had been incorporated successfully into the oxide layer through the hydrolysis of SiF_6_^2−^ anions. This result was consistent with the results of the EDS analysis conducted on spot A and spot B in Figure 1.

### 3.2. Chemical Performance of the Oxide Layers

**Figure 3:**
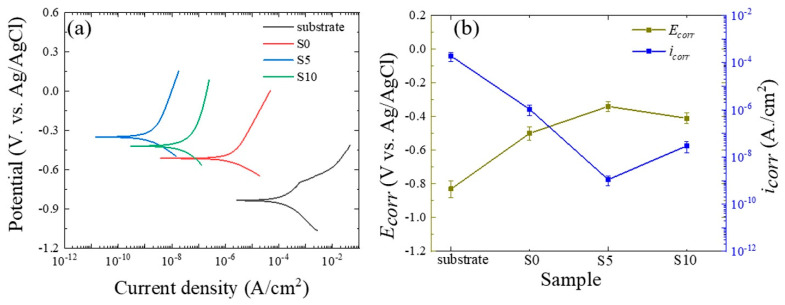
(**a**) PDP curves of the bare substrate and the S0, S5, and S10 samples in a 3.5 wt.% NaCl solution. (**b**) Change of *E_corr_* and *i_corr_* as a function of the SCi concentration.

To determine the influence of SCi concentration on the chemical stability of the samples, PDP curves for the substrate and the S0, S5, and S10 samples are displayed in Figure 3a. The values of the corrosion potential (*E_corr_*) and corrosion current density (*i_corr_*) were derived using the Tafel extrapolation method; the results are presented in Figure 3b. In a typical PDP curve, a positive *E_corr_* value and/or a low *i_corr_* value indicates excellent corrosion resistance, regardless of the material [1]. The *E_corr_* of the substrate was increased in the positive direction by 330, 490, and 420 V in the case of the S0, S5, and S10 samples, respectively. On the other hand, the corresponding values of *i_corr_* for the S0, S5, and S10 samples were 2, 5, and 4 orders of magnitude lower than that of the substrate, respectively. For this reason, the corrosion resistance of the Al alloy was greatly improved by PE treatments in the solutions with SCi additive. According to Figure 1b, a more compact oxide layer was found in the case of the S5 sample, which could prevent the movement of chloride anions into the substrate. For this reason, this sample had the highest chemical stability in the corrosive solution. While it might be expected that the higher thickness obtained in the case of the S10 sample would effectively hinder the ingress of Cl^−^ anions through the structural defects, the sample showed lower chemical stability than the S5 sample. This may be related to the presence of micropores and the higher degree of porosity within the thickness of the S10 sample. The presence of such structural defects, despite the high contents of Si and F, in the S10 sample could be linked to the strong corrosive effect of the electrolyte due to the formation of HF in higher amounts, owing to the complete hydrolysis of SiF_6_^2−^ anions [7]. Thus, we suggest that the high content of HF formed in the case of the S10 sample is the main reason for the formation of a more porous coating as compared to the S5 sample. Here, it is expected that such behavior may be related to the fact that a high content of HF would cause a dissolution of the oxide layer by reducing the pH of the electrolyte. Although the chemical stability of the S10 sample was lower than the S5 sample, it was still higher than S0, confirming that the addition of SCi to SiF_6_^2−^ anion-containing electrolytes could be a promising procedure to improve the chemical stability of Al alloys in a 3.5 wt.% NaCl solution.

**Figure 4:**
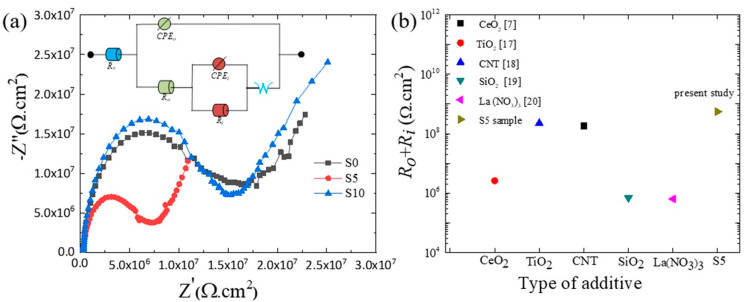
(**a**) Nyquist plots for S0, S5, and S10 samples in a 3.5 wt.% NaCl solution. (**b**) *R_o_* + *R_i_* vs. additive type. The inset in Figure 4a represents the ECM proposed for fitting the EIS results.

To understand the corrosion phenomenon observed in the samples in more detail, the samples were tested using EIS; the results are displayed in Figure 4a in the form of Nyquist plots. In Nyquist plots, the corrosion resistance is linked to the diameter of the capacitive reactance, where a larger diameter of the capacitive arc is an indicator of higher chemical stability [16]. As can be observed in Figure 4a, the capacitive arcs of the S5 and S10 samples were larger than that of the S0 sample, suggesting that the PE treatment greatly reduced the corrosion rate of the S0 sample in electrolytes containing SCi. Similar to the results obtained by the PDP test, the S5 sample showed the largest diameter, which confirms the desirable protective properties obtained due to PE treatment in a solution containing 5 g/L SCi. The EIS results for the S0, S5, and S10 samples were best fitted using the equivalent circuit model (ECM), shown as an inset in Figure 4a. In ECM, *R_o_*/*CPE_o_* and *R_i_*/*CPE_i_* describe the resistance/constant phase element of the outer and inner layer, respectively. A Warburg element (W), which describes diffusion-controlled corrosion, was inserted in the ECM analysis to obtain a better fitting. The obtained results are presented in Table 2. The values of *R_o_* and *R_i_* in the S5 sample were considerably higher than those in other samples, which may be explained by the high-density structure (Figure 1e) that prevents the infiltration of the corrosive anions towards the substrate. Notably, the S5 sample had smaller values of *n_o_* and *n_i_* compared to those of the S0 and S10 samples, implying that uniform oxide layers were successfully obtained [17]. Moreover, the lower values (*Y*_0_ and *Y_i_*) for the S5 sample in comparison to those of the S0 and S10 samples may suggest that the area exposed to the corrosive solution was smaller in the case of the S5 sample due to the clogging of micropores by SiO_2_ and AlF3 compounds [18].

To determine the advantages of SCi anions in increasing the chemical stability of the Al alloy, the values of *R_o_* + *R_i_* for the PE layers formed on Al alloys in addition to the value calculated for the S5 sample were plotted against the additive type included in the electrolyte, as demonstrated in Figure 4b [7,19,20,21,22]. In comparison to other additives, the S5 sample exhibited the highest value of *R_o_* + *R_i_*, confirming that the presence of 5 g/L of SCi in the electrolyte containing SiF_6_^2−^ anions is an effective method for improving the chemical stability of Al alloys.

**Figure 5:**
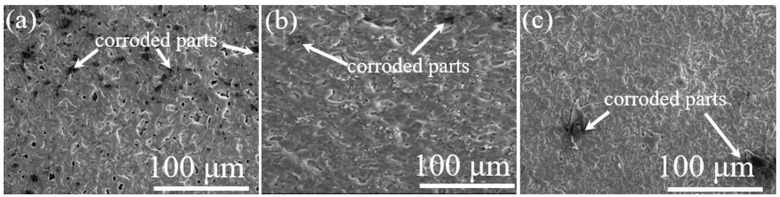
SEM images of the S0, S5, and S10 samples after electrochemical measurements. (**a**) S0 sample, (**b**) S5 sample, and (**c**) S10 sample.

SEM images of the corroded samples are presented in Figure 5 to ascertain the chemical stability of the S0, S5, and S10 samples. Since many structural defects of different sizes were present in the S0 sample, as shown in Figure 1a,d, the corrosive anions (Cl^−^) originating from the 3.5 wt.% NaCl solution could easily penetrate within the oxide layer. Therefore, many corrosion pits and degraded areas were observed on the surface of the S0 sample, as indicated by white arrows in Figure 5a. Such corrosion pits indicate the degradation behavior of the substrate [23]. In contrast, a much lower degradation level was observed on the surfaces of S5 and S10 samples, as shown in Figure 5b,c, and an unaffected surface was noted in the case of the S5 sample, suggesting that desirable chemical stability was achieved in the case of the S5 sample. Therefore, based on the above findings, it can be concluded that PE using an alkaline aluminate–fluorosilicate electrolyte containing SCi is an efficient method for producing dense oxide layers on the surface of 6061 Al alloys suitable for aircraft and aerospace industries, where a good combination of low weight and high corrosion resistance (chemical stability) is considered to be necessary for such applications.

### 3.3. Mechanism Underlying the Promotion of SiF_6_^2−^ Anion Hydrolysis

**Figure 6:**
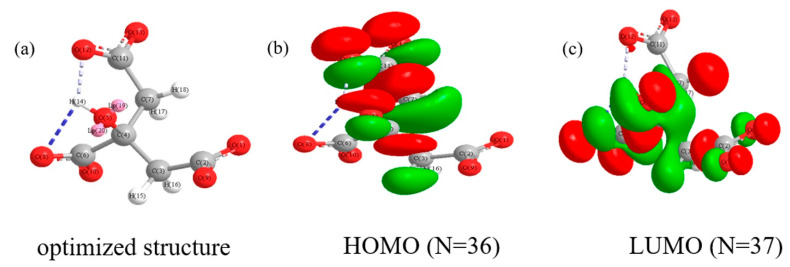
(**a**) Optimized molecular computed structure of citrate anion. Frontier molecular orbital density distribution of (**b**) HOMO (N = 36) and (**c**) LUMO (N = 37).

To comprehend the role of SCi in triggering the hydrolysis of SiF_6_^2−^ anions and their impact on the formation of highly compact oxide layers, computational analysis was conducted. The HOMO and LUMO of the citrate anion are presented in Figure 6. In general, the HOMO and LUMO values describe the capability of molecules or anions to donate and accept electrons, respectively [24,25,26,27]. The quantum parameters, including *A*, *I*, *η*, *σ*, *χ*, and Δ*N*, were calculated by Equations (3)–(8) and the results are listed in Table 3.

(3) $$I=-E_{HOMO}$$

(4) $$A=-E_{LUMO}$$

(5) $$\chi =\frac{I+A}{2}$$

(6) $$\eta =\frac{I-A}{2}$$

(7) $$\sigma =\frac{1}{\eta }$$

(8) $$∆N=\frac{\left(\chi_{Mg}\;-\;\chi_{mol}\right)}{2\left(\eta_{Mg}+\eta_{mol}\right)}$$

For the calculation of Δ*N* in Equation (8), theoretical values of *χ_Al_* (3.23 eV/mol) and *η_Al_* (2.77 eV mol) for aluminum were considered [28]. As reported earlier [5,24,25,26,27,28], the binding capability of molecules or anions with metal and coating surfaces usually increases with a high *E_HOMO_* value and lower *E_LUMO_* values. Thus, based on the results shown in Table 3, SCi anions could bind strongly with the surface of the substrate or coating by donating electrons from their HOMO to the empty d-orbitals of the Al metal or metal oxides. The high reactivity of SCi anions and the formation of the adsorption layer on the substrate surface were approved by the value of ∆*N*, which reflects their ability to donate electrons [28]. The parameters of *η* and *σ* confirmed the high reactivity of SCi anions during the initial stages of PE. Accordingly, SCi anions can be physisorbed and or chemisorbed on the surface of the substrate during the initial stages of the PE process, which is correlated to the negative charges of these anions as well as their capability to form stable complexes through the interaction between their HOMO and the valence d-orbital of Al^3+^ ions. Here, SCi anions can be considered as a Lewis base due to their ability to substitute the six monodentate water molecules of Al(H_2_O)_6_^3+^. Thus, an electrochemical double layer (EDL) is formed on the S5 and S10 surfaces due to the adsorption of SCi anions [5]. The structure of the complex ligand can be described by Equation (9).

Al^3+^ + (C_6_H_5_O_7_)^3−^   →   [Al (C_6_H_5_O_7_)] (9)

**Figure 7:**
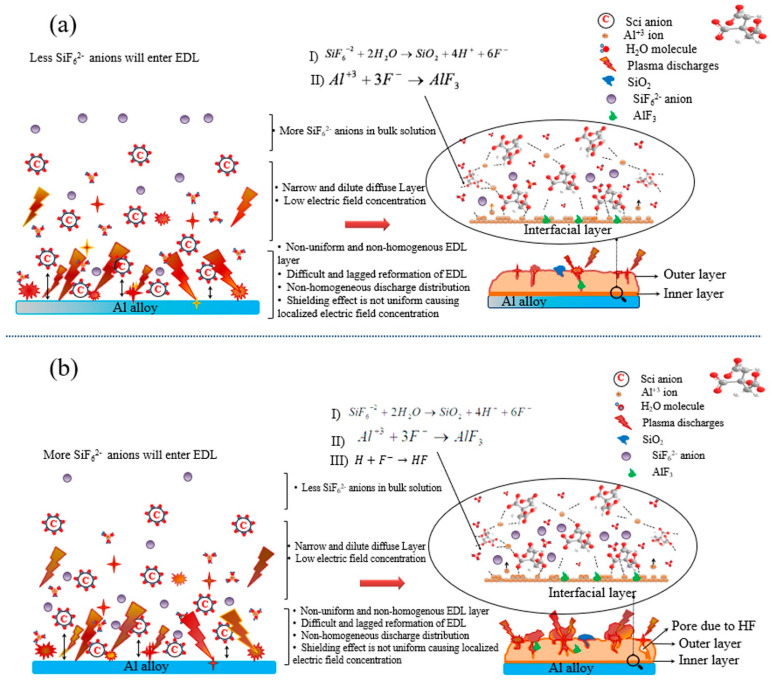
Schematic illustration showing the formation of the EDL as well as the hydrolysis reaction of SiF_6_^2−^ anions on the surface of the (**a**) S5 sample and (**b**) S10 sample.

As reported earlier [5,29,30,31], the presence of SCi anions could improve the distribution of other additives in the electrolyte, which would help to form a homogenous EDL on the substrate surface. To understand the formation of the oxide layers in the case of the S5 and S10 samples, schematic illustrations of the formation of the EDL as well as the hydrolysis reaction of SiF_6_^2−^ anions are shown in Figure 7. Under plasma conditions and at the lower concentration of SCi (5 g/L; Figure 7a), a small amount of SiF_6_^2−^ reached the surface of the EDL to pick up electrons that were moving across this layer from SCi. Thus, the occurrence of the reactions in Equations (1) and (2) were restricted to some extent. Since the F^−^ anions resulting from Equation (2) are known to be the smallest anion, they could reach the inner parts of the oxide layer. Thus, an inner layer rich with AlF_3_ was produced. In contrast, a high concentration of SCi attracted more SiF_6_^2−^ anions to move through the thick EDL (Figure 7b). Therefore, it is thought that the participation of SiF_6_^2−^ anions in the formation of the oxide layer in the case of the S10 sample would be greater when compared to that in the S5 sample. As a result, an increased number of SiF_6_^2−^ anions were subjected to hydrolysis during the short coating time used in the present work. This assumption is in line with the EDS results in Table 1. However, since HF would be formed in higher amounts in the case of the S10 sample, as discussed above, the compactness of the S10 sample was somehow lower than that of the S5 sample. This finding justifies the importance of the optimization of SCi concentration during PE to obtain a conformal oxide layer with desirable chemical stability in the corrosive solution. Thus, two reasons may be responsible for improving the chemical stability of the coatings made in the SCi-containing electrolytes. First, the compact structure obtained in the case of the S5 sample could significantly restrict the movement of the corrosive anions towards the substrate, leading to the improvement in the corrosion-protection properties of the 6061 Al alloy coated by PE. Second, the incorporation of stable oxide (SiO_2_) could also effectively suppress the penetration of the corrosive anions into the Al alloy substrate. As reported earlier [32,33,34], the incorporation of stable oxide particles, such as SiO_2_, TiO_2_, and MoO_2_, into the oxide layer on 6061 Al alloy via PE can significantly improve the chemical stability of Al alloy in 3.5 wt.% NaCl solution.

## 4. Conclusions

In this work, we exploited the hydrolysis reaction of SiF_6_^2−^ anions induced by SCi in addition to controlling the porosity in the plasma inorganic layers through a novel process that involved regulating the incorporation of silicon and fluorine-based compounds. The results of the present study can be summarized as follows:

(1) PE treatment in solutions containing SCi gave rise to relatively compact structures with minimum sizes of micropores.
(2) A thick EDL tended to be formed on the substrate surface during the initial stage of the PE process conducted in a solution containing SCi additive.
(3) The hydrolysis of SiF_6_^2−^ anions transferred through the EDL was found to be affected by the thickness of the EDL.
(4) A controlled hydrolysis of SiF_6_^2−^ anions was found in the case of the S5 sample treated in a solution containing 5 g/L of SCi, which resulted in the formation of a very dense oxide layer on its surface.
(5) The S5 sample exhibited the lowest value of *i_corr_*, the highest value of *E_corr_*, and the highest impedance. Thus, the chemical stability of this sample in 3.5 wt.% NaCl solution was superior to the other samples.

## Figures and Tables

**Table 1:** Results of EDS conducted on the surface of S0, S5, and S10 samples.

Sample	Al (wt.%)	O (wt.%)	Si (wt.%)	F (wt.%)	C (wt.%)
S0	47.05	45.27	1.16	6.52	-
S5	27.72	38.41	6.50	15.94	11.43
S10	27.20	25.96	8.98	20.15	17.71

**Table 2:** Results of the EIS tests of the S0, S5, and S10 samples immersed for 6 h in a 3.5% NaCl solution.

Sample	*R_s_* × 10^−3^ (Ωcm^2^)	*R_o_* (MΩcm^2^)	*CPE-n_o_*	*CPE-Y_o_* × 10^−12^ (S.s^n^.cm^−2^)	*R_i_* (MΩcm^2^)	*CPE-n_i_*	*CPE-Y_i_* × 10^−9^ (S.s^n^.cm^−2^)	*W* × 10^−6^ (S*S^1/2^)
S0	1.34 ± 1.05	0.273 ± 0.03	0.78 ± 0.03	18.4 ± 2.77	7.77 ± 0.97	0.48 ± 0.12	6.52 ± 3.64	0.326 ± 0.08
S5	3.58 ± 2.22	4.57 ± 1.13	0.91 ± 0.02	1.90 ± 0.77	550 ± 38.0	0.72 ± 0.17	0.172 ± 0.08	9.72 ± 1.43
S10	7.60 ± 3.48	0.822 ± 0.02	0.83 ± 0.01	5.25 ± 2.19	21.2 ± 3.42	0.56 ± 0.14	6.03 ± 4.11	3.98 ± 0.93

**Table 3:** Quantum chemical parameters calculated for SCi anions.

Anion	*E_HOMO_* (eV)	*E_LUMO_* (eV)	∆E (eV)	*A*	*I*	*χ*	*η*	∆*N*	σ
SCi	−12.356	−10.807	1.549	10.807	12.356	11.581	0.774	−1.178	1.291

## Data Availability

The raw/processed data required to reproduce these findings cannot be shared at this time as the data also form part of an ongoing study.
